# Case Report: Sporadic Creutzfeldt–Jakob disease initially misdiagnosed as ischemic stroke: a diagnostic challenge and biomarker-based clarification

**DOI:** 10.3389/fncel.2026.1938627

**Published:** 2026-10-16

**Authors:** Wenjie Hao, Xuan Zhou, Meixia Wang, Wenming Yang, Ting Cheng

**Affiliations:** 1 The First Affiliated Hospital of Anhui University of Chinese Medicine, Hefei, Anhui, China; 2 Brain Disease Research Institute, Anhui University of Chinese Medicine, Hefei, China; 3 Key Laboratory of XinAn Medicine, Ministry of Education, Hefei, China

**Keywords:** case report, cerebrospinal fluid biomarkers, cortical ribboning, sporadic Creutzfeldt–Jakob disease (sCJD), stroke misdiagnosis

## Abstract

**Background:**

Sporadic Creutzfeldt–Jakob disease (sCJD) is a rare and uniformly fatal prion disorder characterized by rapidly progressive dementia and heterogeneous neurological manifestations. Early sCJD may be misinterpreted as cerebrovascular disease, particularly when focal neurological deficits, unilateral diffusion abnormalities, and vascular risk factors coexist.

**Case presentation:**

We report a 76-year-old man with a history of hypertension who initially presented with dizziness and gait instability, followed by progressive right-sided weakness, and was initially diagnosed with ischemic stroke in the setting of intracranial arterial stenosis. Despite antiplatelet and vascular management, the patient experienced rapid cognitive decline, psychiatric disturbances, aphasia, and progressive hemiparesis. Follow-up MRI demonstrated progressive, non-territorial cortical ribbon-like diffusion restriction predominantly involving the left insular, parietal, frontal, and temporal cortices. CSF analysis revealed markedly elevated total tau (4553.50 pg/mL; laboratory-reported cutoff: < 399 pg/mL), phosphorylated tau (66.93 pg/mL; laboratory-reported cutoff: < 50 pg/mL), and 14-3-3γ protein (50515 AU/mL; laboratory-reported cutoff: < 20,000 AU/mL). Autoimmune encephalitis antibodies and metagenomic next-generation sequencing were negative. PRNP genetic testing detected no pathogenic variant in the patient and his son. EEG showed diffuse slowing without periodic sharp wave complexes. According to the 2017 International CJD Surveillance Network criteria, the diagnosis was classified as probable sCJD on the basis of rapidly progressive cognitive impairment, two clinical features (documented cerebellar ataxia and pyramidal signs), characteristic MRI abnormalities, and positive CSF 14-3-3; the typical EEG criterion was absent and RT-QuIC was not performed.

**Conclusion:**

This case highlights the diagnostic challenge of early sCJD initially misdiagnosed as ischemic stroke in the presence of coexisting vascular pathology. Recognition of non-vascular diffusion patterns, careful reassessment of atypical clinical progression, and adjunctive CSF biomarker assessment may facilitate timely diagnostic reconsideration and help avoid unnecessary interventions.

## Background

Creutzfeldt–Jakob disease is a fatal neurodegenerative disorder caused by the conformational conversion of normal prion protein (PrP^C) into its pathological isoform (PrP^Sc) (Shkundina and Ter-Avanesyan, 2007). The sporadic form accounts for approximately 85% of cases, with an annual incidence of 1–2 cases per million population (Zerr et al., 2009). Epidemiological data on CJD in Asian populations remain relatively limited. Available surveillance data indicate that sporadic CJD is the predominant form of human prion disease in Asia, including mainland China (Gao et al., 2024; Shi et al., 2022; Shi et al., 2023). The classical clinical phenotype includes rapidly progressive dementia (RPD), myoclonus, cerebellar dysfunction, pyramidal and extrapyramidal signs, and eventual akinetic mutism (Geschwind, 2016). However, early manifestations are frequently nonspecific and may resemble stroke, autoimmune encephalitis, psychiatric disorders, or metabolic encephalopathy (Hermann et al., 2021).

The introduction of diffusion-weighted imaging (DWI) and CSF biomarkers such as 14-3-3 protein and tau protein has substantially improved premortem diagnosis (Green, 2019). More recently, revised international surveillance criteria incorporating MRI cortical ribboning and RT-QuIC testing have further increased diagnostic sensitivity without compromising specificity (Watson et al., 2022). Here, we describe a case of sCJD initially misdiagnosed as ischemic stroke in the setting of intracranial arterial stenosis (Supplementary Video 1 and Supplementary Data Sheet 1), illustrating a critical diagnostic dilemma in the evaluation of subacute focal neurological deficits.

## Case presentation

### Initial presentation

A 76-year-old Han Chinese man presented with a 2-month history of dizziness and gait instability, followed by progressive right-sided weakness for one week. He had a history of hypertension for more than 10 years and had been taking valsartan 80 mg orally every morning on a regular basis, with home blood pressure generally maintained at approximately 120–130/70–80 mmHg. He had no history of dyslipidemia, hypercholesterolemia, or diabetes mellitus and was taking no other regular medication before the onset of the present illness. He had no prior history of stroke or dementia. His family history was negative for cerebrovascular disease and for known hereditary neurological or prion disease. Two months before admission, he developed imbalance and experienced a fall at home. At the outside hospital, the initial neurological assessment documented gait and truncal ataxia and impaired rapid alternating movements, consistent with cerebellar dysfunction. Brain MRI was reported as showing a presumed left parietal subcortical ischemic lesion and chronic ischemic white matter changes. After the initial diagnosis of ischemic stroke, treatment at the outside hospital consisted of enteric-coated aspirin 100 mg orally once daily, rosuvastatin calcium 10 mg orally nightly, valsartan 80 mg orally every morning, butylphthalide soft capsules (two capsules orally three times daily), and ligustrazine hydrochloride 80 mg diluted in 250 mL of 0.9% sodium chloride intravenously once daily.

### Vascular confirmation and diagnostic anchoring

Subsequent cerebral angiography revealed stenosis of the left middle cerebral artery and severe stenosis of the A1 segment of the anterior cerebral artery. The working diagnosis was ischemic stroke secondary to intracranial atherosclerosis. At this stage, the diagnosis appeared consistent with the imaging findings and vascular risk profile, reinforcing diagnostic anchoring. Because the anticipated procedural risk was considered to outweigh the expected benefit, no endovascular or surgical revascularization procedure (including angioplasty or stenting) was performed for the intracranial stenoses. The vascular lesions were managed conservatively with medical therapy, including the above antiplatelet and circulation-oriented treatment.

### Clinical progression

Although vascular treatment was initiated, the patient showed continuous neurological deterioration rather than stabilization or a stepwise pattern typical of recurrent cerebrovascular events. Over several weeks, he developed progressive cognitive impairment, psychiatric disturbances, speech impairment evolving to aphasia, and worsening right hemiparesis. Neurological examination demonstrated reduced responsiveness, increased upper limb tone, bilateral ankle clonus, and right-sided weakness (Medical Research Council grade III). This clinical course was atypical for a stabilized ischemic stroke.

### Neuroimaging evolution

Serial neuroimaging demonstrated dynamic evolution from initially presumed vascular pathology to a non-territorial cortical diffusion pattern. Brain MRI with DWI performed approximately 2 month before admission was reported as showing a recent presumed left parietal subcortical ischemic lesion, along with bilateral frontal lobe, basal ganglia, periventricular white matter, and centrum semiovale ischemic changes consistent with chronic small vessel disease (Figures 1A–C). Subtle cortical ribboning may have been present in retrospect but was not recognized at the time.

**FIGURE 1:**
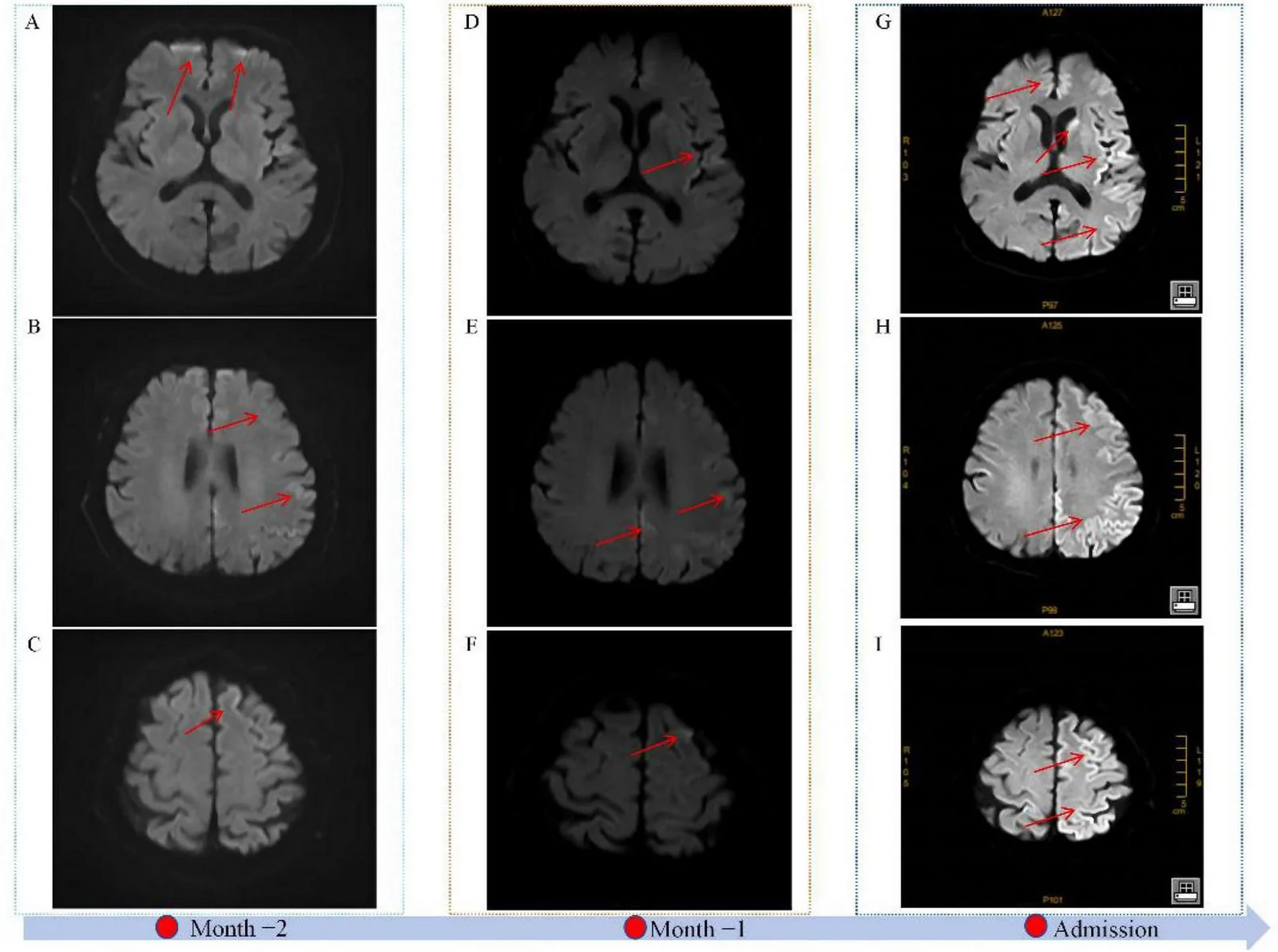
DWI imaging of MRI at different time points. Serial axial diffusion-weighted imaging (DWI) demonstrates progressive cortical abnormalities. Arrows indicate cortical ribboning. **(A–C)** Month -2: Initial DWI was reported as showing a presumed left parietal subcortical ischemic lesion, with subtle cortical abnormality appreciable in retrospect. **(D–F)** Month -1: Follow-up DWI revealed emerging gyriform hyperintensity involving the left insular and parietal cortices. **(G–I)** Admission: Repeat DWI demonstrated extensive cortical ribbon-like hyperintensity involving the left frontal, parietal, temporal, and insular cortices.

A follow-up MRI performed approximately 1 month before admission revealed subtle abnormal DWI hyperintensity involving the left insular and parietal cortices (Figures 1D–F). The findings were initially interpreted as possible ischemic change in the context of pre-existing white matter disease and age-related cerebral degeneration.

Head CTA performed during the same early diagnostic period demonstrated intracranial arterial stenosis without a perfusion deficit sufficient to explain the evolving cortical abnormalities (Figure 2). Repeat MRI performed at our institution after admission demonstrated extensive gyriform hyperintensity along the left frontal, parietal, temporal, and insular cortices on diffusion-weighted imaging, with corresponding low signal intensity on apparent diffusion coefficient (ADC) maps, confirming true diffusion restriction rather than T2 shine-through (Figures 1G–I). Cortical ribboning (gyriform restricted diffusion) is characteristic of sCJD and typically does not conform to vascular territories Vitali et al., 2011; Young et al., 2005. The progressive, non-territorial cortical diffusion pattern strongly argued against a purely ischemic mechanism and raised substantial suspicion for sCJD in the appropriate clinical context.

**FIGURE 2:**
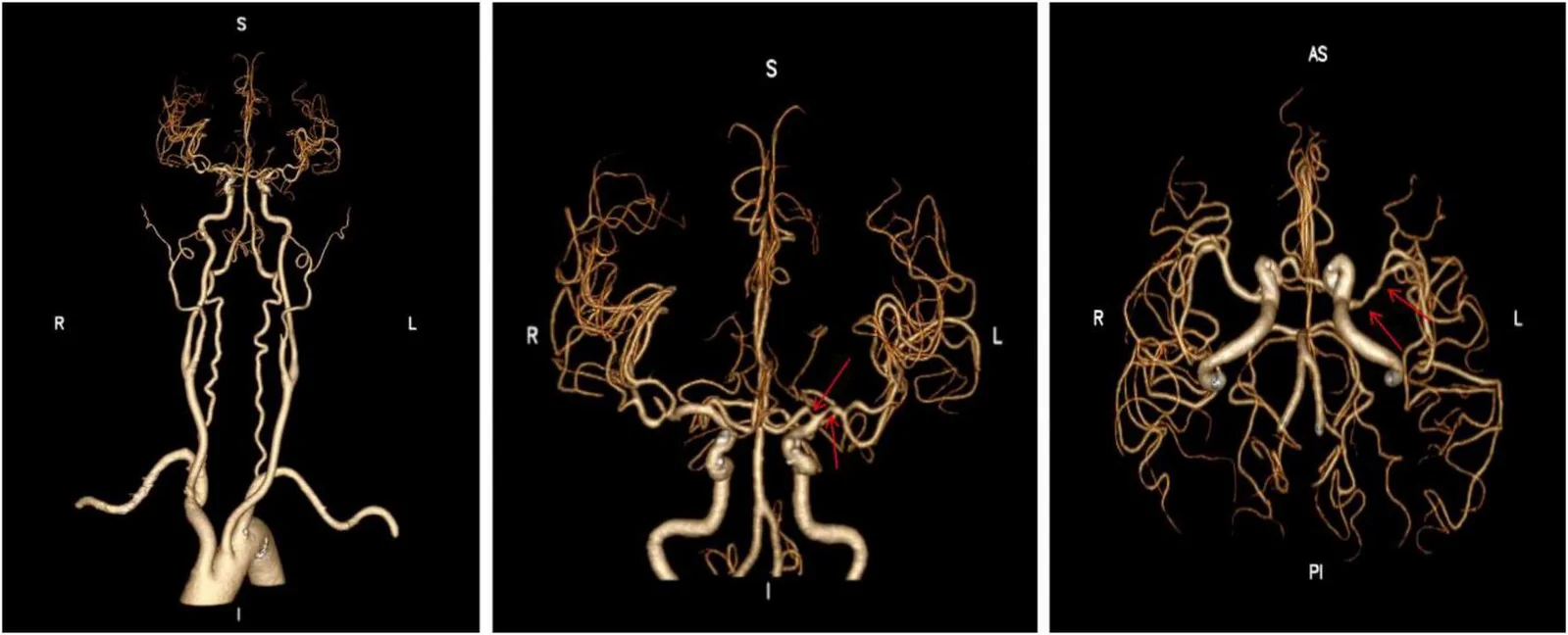
Head CTA imaging. Head computed tomography angiography (CTA) demonstrates stenosis of the left middle cerebral artery and severe stenosis of the A1 segment of the left anterior cerebral artery. Arrows and asterisks indicate the stenotic vascular segments.

### Diagnostic evaluation and cerebrospinal fluid findings

EEG demonstrated diffuse slowing without periodic sharp wave complexes. Lumbar puncture revealed elevated CSF protein (765.87 mg/L; reference range: 150–450 mg/L). Routine and biochemical CSF testing showed a cell count, glucose concentration, and chloride concentration within their respective reference ranges. CSF was collected by lumbar puncture at 11:00 on September 16, 2025. Biomarker testing was performed by an external laboratory (Keruidi Medical Laboratory, Hangzhou, China). The CSF Alzheimer biomarker panel (Aβ40, Aβ42, total tau [t-tau], and phosphorylated tau [p-tau]) was measured by ELISA; t-tau and p-tau were assayed using the hTau total ELISA and phosphoTAU ELISA, respectively, distributed by IBL International GmbH (Hamburg, Germany), with absorbance measured on a Multiskan FC microplate photometer (Thermo Fisher Scientific). CSF 14-3-3γ was quantified using the CircuLex 14-3-3 Gamma ELISA Kit (catalog no. CY-8082; MBL, Japan), a quantitative sandwich ELISA, with absorbance measured using a SUNRISE microplate absorbance reader (Tecan Austria GmbH, Grödig, Austria). Further analysis demonstrated markedly elevated total tau (4553.50 pg/mL; laboratory-reported cutoff: < 399 pg/mL), phosphorylated tau (66.93 pg/mL; laboratory-reported cutoff: < 50 pg/mL), and 14-3-3γ protein (50,515 AU/mL; laboratory-reported cutoff: < 20,000 AU/mL), while the autoimmune encephalitis antibody panel and CSF metagenomic next-generation sequencing for infectious pathogens were negative. These interpretive cutoffs were those reported by the external testing laboratory for the assays used. PRNP genetic testing was performed in both the patient and his son, and no pathogenic variants were detected in either individual (Supplementary Data Sheet 2). RT-QuIC could not be performed because the assay was unavailable at our institution. Several external/reference testing centers were contacted regarding specimen referral, but none was able to provide RT-QuIC testing for this patient at that time.

According to the 2017 International CJD Surveillance Network criteria9, the patient fulfilled criterion I (rapidly progressive cognitive impairment) and two criterion II features: cerebellar dysfunction, documented at the initial outside-hospital assessment by gait and truncal ataxia and impaired rapid alternating movements, and pyramidal features, represented by progressive right hemiparesis, increased upper-limb tone, and bilateral ankle clonus. Myoclonus, visual symptoms, and akinetic mutism were not documented. The MRI criterion was fulfilled by restricted diffusion involving at least two qualifying cortical regions (left parietal and temporal cortices) on DWI with corresponding ADC reduction. CSF 14-3-3γ was positive (50,515 AU/mL; laboratory-reported cutoff < 20,000 AU/mL) within a disease duration of less than 2 years. The EEG criterion was not fulfilled because the EEG showed diffuse slowing without periodic sharp wave complexes. RT-QuIC was unavailable and therefore not performed. Routine investigations did not identify a more plausible alternative diagnosis. Thus, the case met criteria for probable sCJD. Total tau and p-tau were considered supportive markers of neuronal injury but were not used as core criteria for classification.

Key diagnostic turning points over the disease course are summarized in Table 1. The patient was discharged with supportive care as neurological decline continued. After discharge, the patient continued to deteriorate neurologically over the following 6 months, with progressive loss of communication and increasing dependence in activities of daily living, and died 6 months later.

**TABLE 1:** Key diagnostic turning points.

Time	Clinical status	Key findings	Diagnostic implication
Month -2	Dizziness; gait and truncal ataxia; impaired rapid alternating movements	MRI reported as showing a presumed left parietal ischemic lesion	Initial diagnosis: ischemic stroke
Month -1	Persistent symptoms	Angiography: left MCA + ACA stenosis	Reinforced vascular diagnosis
Admission	Rapid cognitive decline, psychiatric symptoms, hemiparesis	–	Atypical for stabilized stroke
Hospital Day 3	Neurological deterioration	MRI: cortical ribbon-like DWI restriction	Suspicion of non-vascular etiology
Hospital Day 5	Progressive encephalopathy	CSF protein elevated	Consider inflammatory/prion disease
Hospital Day 10	Severe RPD	CSF: 14-3-3γ 50,515 AU/mL; t-tau 4553.50 pg/mL	Diagnosis revised to probable sCJD

RPD, rapidly progressive dementia; MCA, middle cerebral artery; ACA, anterior cerebral artery; MRI, magnetic resonance imaging; DWI, diffusion-weighted imaging; CSF, cerebrospinal fluid; 14-3-3, 14-3-3 protein; t-tau, total tau protein; sCJD, sporadic Creutzfeldt–Jakob disease.

## Discussion

### sCJD initially misdiagnosed as ischemic stroke: a diagnostic dilemma

Although sporadic Creutzfeldt–Jakob disease (sCJD) typically presents with rapidly progressive dementia, early focal deficits may mimic acute stroke (Appleby et al., 2009; Geschwind, 2016). In the present case, the clinical course was not consistent with a classic hyperacute stroke-like onset. Rather, the patient developed subacute dizziness and gait instability followed by progressive right-sided weakness and rapid multidomain neurological deterioration. Therefore, we reframed this case as sCJD initially misdiagnosed as ischemic stroke, rather than as a typical stroke-like presentation.

Initial imaging was interpreted as showing a presumed ischemic lesion, and the presence of vascular risk factors and intracranial arterial stenosis reinforced a vascular diagnosis. However, several features argued against a purely ischemic mechanism: the neurological decline was continuous and progressive rather than sudden or stepwise; the diffusion abnormalities evolved beyond a vascular territory; and serial MRI showed progressive cortical ribboning with ADC reduction. These features prompted reconsideration of the initial diagnosis.

A key distinguishing feature was the dynamic imaging evolution. Serial MRI demonstrated progression to extensive, non-territorial cortical diffusion restriction with corresponding ADC reduction, supportive of sCJD in the appropriate clinical context. In patients with presumed stroke who show atypical features, especially ongoing cognitive decline, psychiatric symptoms, aphasia, and progressive focal deficits despite appropriate vascular management, repeat MRI should be performed and carefully reviewed for cortical ribboning and non-territorial diffusion abnormalities. This case therefore highlights the importance of careful radiological reassessment and adjunctive CSF biomarker assessment when the clinical course is incompatible with ischemic stroke.

### MRI as a diagnostic turning point

DWI is highly sensitive for sCJD (Young et al., 2005). Cortical ribboning and basal ganglia hyperintensity are typical imaging features, with reported sensitivity approaching 90–96% (Vitali et al., 2011). In contrast to ischemic infarction, lesions in sCJD do not conform to vascular territories, diffusion abnormalities may spread or fluctuate, and bilateral or patchy cortical involvement is common. The 2017 revised international criteria incorporated MRI cortical ribboning because of its high diagnostic utility (Watson et al., 2022).

Recent studies further support the diagnostic value of DWI-based MRI criteria in suspected sCJD. A diffusion MRI criterion based on involvement of at least one positive brain region among cortical and deep gray matter structures has been proposed and evaluated, demonstrating improved sensitivity for prion disease detection (Bizzi et al., 2020). Newly proposed MRI criteria for sCJD diagnosis have also been assessed, further emphasizing the importance of systematic MRI interpretation in suspected cases (Barber et al., 2024). Recognition of these imaging features represented the diagnostic turning point in this case.

### CSF biomarkers: panel-based interpretation

A structured meta-analysis reported a pooled sensitivity of 92% and specificity of 80% for CSF 14-3-3 testing in patients with suspected sCJD; however, diagnostic performance varies with clinical context, disease stage, and molecular subtype (Muayqil et al., 2012). However, specificity is limited, as elevations may occur in stroke, encephalitis, and hypoxic injury (Otto et al., 2002). Total tau levels are typically markedly elevated in sCJD and improve specificity when interpreted in conjunction with 14-3-3 protein (Otto et al., 2002). Marked elevation of total tau (for example, > 1200 pg/mL) has been reported to improve diagnostic specificity when interpreted together with 14-3-3 protein positivity. In our patient, total tau exceeded 4500 pg/mL, strongly supporting prion-mediated neuronal damage. RT-QuIC has emerged as a highly specific diagnostic assay for prion disease (McGuire et al., 2012). We acknowledge the absence of RT-QuIC as an important limitation of this case. At the time of evaluation, the assay was unavailable at our institution, and despite contacting several external/reference testing centers, RT-QuIC could not be obtained for this patient. Because neuropathological confirmation was unavailable, the diagnosis remained probable rather than definite; the absence of RT-QuIC was an additional diagnostic limitation. The diagnosis was supported by the combination of clinical progression, characteristic MRI findings, and positive CSF 14-3-3 within the established diagnostic framework.

### Differential diagnosis in rapidly progressive dementia

Rapidly progressive dementia constitutes a neurological emergency requiring exclusion of treatable causes. Differential diagnoses include autoimmune encephalitis (Graus et al., 2016), viral encephalitis, paraneoplastic encephalopathy, and toxic–metabolic disorders. In the present case, CSF routine and biochemical analysis showed no pleocytosis and normal glucose and chloride levels; the autoimmune encephalitis antibody panel was negative; and CSF metagenomic next-generation sequencing did not detect an infectious pathogen. These findings provided no evidence for an infectious or autoimmune encephalitic etiology. Gyriform diffusion restriction may also occur in seizures, hypoxic–ischemic injury, and metabolic disturbances; these findings should therefore be interpreted in the overall clinical context (Vitali et al., 2011). In this patient, postictal change was considered less likely because there was no clear history of prolonged seizure activity or focal status epilepticus, and EEG did not show epileptiform discharges. Hypoxic–ischemic injury was also unlikely because there was no history of cardiac arrest, severe systemic hypotension, or global hypoxic insult. Metabolic encephalopathy was considered less likely given the progressive focal cortical MRI evolution, absence of a compatible systemic metabolic disturbance, and the CSF biomarker profile indicating substantial neuronal injury when interpreted together with the clinical and imaging evolution.

### Epidemiological context in Asia and China

Human prion diseases are rare, with an estimated annual mortality of approximately 1–2 cases per million population, but epidemiological ascertainment in Asia remains uneven. Long-term surveillance has been established mainly in Japan, South Korea and China, while under-recognition remains a concern in settings without systematic surveillance (Gao et al., 2024). Chinese national surveillance has reported more than 2,100 diagnosed prion disease cases, with sCJD representing approximately 88.7% and genetic prion diseases approximately 11.3% (Shi et al., 2022). sCJD and other prion diseases have been reported across multiple Chinese provincial-level administrative divisions; an analysis of 1,970 referred cases from 2006 to 2019 included 344 hospitals across 29 provincial-level administrative divisions and demonstrated substantial geographic variation in referral and case recognition, partly associated with surveillance coverage (Shi et al., 2023). Chinese genetic prion disease also shows geographic heterogeneity: fatal familial insomnia and T188K- and E200K-associated genetic CJD are among the more frequently identified forms, and earlier surveillance noted apparent clustering in some provinces, including Henan, although differential surveillance intensity may contribute to this pattern (Shi et al., 2015). These data indicate that both sporadic and genetic prion diseases are distributed across China, but observed geographic differences should be interpreted in the context of referral patterns and surveillance structure.

### Clinical implications

This case underscores several important clinical lessons. Coexisting vascular pathology does not exclude neurodegenerative diseases. Rapid multi-domain neurological decline despite adequate stroke management should prompt diagnostic reassessment. Subtle unilateral cortical ribboning may be overlooked or misinterpreted as ischemic change at an early stage. CSF biomarker testing may provide useful adjunctive evidence when rapidly progressive dementia is suspected and should be interpreted together with the clinical course, MRI, EEG, and exclusion of alternative diagnoses. Timely recognition may prevent unnecessary escalation of vascular interventions and facilitate appropriate counseling.

## Conclusion

Sporadic CJD may be initially misdiagnosed as ischemic stroke, particularly in elderly patients with vascular risk factors and coincident vascular imaging abnormalities. Recognition of non-territorial cortical diffusion restriction and integrated, appropriately cautious interpretation of CSF biomarkers may facilitate earlier diagnostic reconsideration and help avoid misdirected management.

## Data Availability

The original contributions presented in the study are included in the article/Supplementary material, further inquiries can be directed to the corresponding authors.
