# Supplementary material for: Case Report: Sporadic Creutzfeldt–Jakob disease initially misdiagnosed as ischemic stroke: a diagnostic challenge and biomarker-based clarification
Source: Front Cell Neurosci. 2026 Oct 16;20:1938627. doi: 10.3389/fncel.2026.1938627 (PMC13719361; doi:10.3389/fncel.2026.1938627)
Supplement: Supplementary file 1 [file Data_Sheet_1.pdf]

患者知情同意告知书

（Patient Informed Consent Notice）

患者姓名(Patient Name) 张明家 (Mingjia Zhang) 性别(Gender) 男 (male) 年龄(Age) 76 岁(Year)

身份证号(ID Number)

3	4	1	1	2	5	1	9	4	9	0	1	0	1	3	8	5	4

患者对其资料发表于国内外学术期刊的知情同意书。

本人同意我的资料出现在学术刊物和相关出版物。我已看过文中所用图片并且了解了发表的内容。

我已获知：

1、我的名字不会被公开，但我也理解不能保证完全保密。

2、我的资料会发表于全球性的网站和期刊上，印刷版本和网络版本会供医生、媒体、大众阅读。

3、我的资料不会被用于商业用途。

4、我知道这些影像、检查及检验等资料会用于向医学界或公众宣传我所患疾病的特征性表现、治疗方法及效果、病程进展、诊治难点和其他相关问题。

The patient's informed consent form for their information to be published in domestic and foreign academic journals.

I agree that my information appears in academic and related publications. I have reviewed the images used in the article and understand the content of the publication.

I understand that:

1. My name will not be made public, but I do understand that complete confidentiality cannot be guaranteed.

2. My information will be published on global websites and journals, and printed and online versions will be available for doctors, media, and the public to read.

3. My information will not be used for commercial purposes.

4. I understand that these imaging, examinations and tests will be used to inform the medical community or the public about the characteristic manifestations, treatment methods and effects, progression of the disease, diagnosis and treatment difficulties and other related issues.

患者/监护人签字(Signature of patient/guardian) 张胜芳 (Shengfang Zhang)

签字日期(Signing date) 25 日(day) 12 月(month) 2025 年(year)

联系电话(Contact phone number) 13709604679

医生签名(Signature of doctor) 郝文杰 (Wenjie Hao)

签字日期(Signing date) 25 日(day) 12 月(month) 2025 年(year)

（知情同意书一式两份，医生患者各一份）

(This informed consent form is in duplicate, one for the doctor and one for the patient)
