# Supplementary material for: Case Report: Sporadic Creutzfeldt–Jakob disease initially misdiagnosed as ischemic stroke: a diagnostic challenge and biomarker-based clarification
Source: Front Cell Neurosci. 2026 Oct 16;20:1938627. doi: 10.3389/fncel.2026.1938627 (PMC13719361; doi:10.3389/fncel.2026.1938627)
Supplement: Supplementary file 2 [file Data_Sheet_2.pdf]

科睿迪医学
CRED DIAGNOSTICS

科睿迪医学

PRNP 基因检测

基本信息

1.受检者			
姓名：张明家	性别：男	年龄：76	联系方式：-
**2.临床信息**			
诊断：-			
临床症状及家族史：头晕，行走不稳 2 月有余			
**3.样本及送检信息**			
样本编号：A00KRD034281	样本类型：☑EDTA 抗凝血	住院号/病历号：-	
送检医院：安徽中医药大学第一附属医院	科室：脑病科	床号：9 床	
采样日期：-	接收日期：2025-09-25	报告日期：2025-09-30	
**4.检测项目信息**			
检测项目：*PRNP* 基因测序			
检测方法：Sanger 测序			

检测结果

检测结论
在检测范围内，未检出致病或疑似致病变异<br>受检者检测到 129 位氨基酸多态性为 M/M 型，219 位氨基酸多态性为 E/E 型

检测结果统计

主要检测结果：与症状可能相关的变异

基因/转录本	基因亚区	突变信息	突变类型	致病性分类	遗传方式	疾病/表型	RS 号	文献
-	-	-	-	-	-	-	-	-

备注：
1.数据解读规则参考美国医学遗传学和基因组学学院（American College of Medical Genetics and Genomics，ACMG）相关指南。变异命名参照 HGVS 建议的规则给出（http://www.hgvs.org/mutnomen/）。
2.遗传方式：AD，常染色体显性遗传；AR，常染色体隐性遗传；XLD，X 染色体显性遗传；XLR，X 染色体隐性遗传；-，遗传方式未明。

本次检测，在受检者中未检出致病或疑似致病变异。

检验者：郭成忠　　报告者：吴引东

杭州科睿迪智检医学检验实验室 报告专用章 2025-9-30

检测基因列表

检测基因
PRNP

Sanger 测序结果

注：由于测序采用正向测序或反向测序，结果显示的碱基有可能为被检测碱基的反向互补序列，如：c.163G>A，峰图可显示为 G>A 或其反向互补序列 C>T。

分析样本	分析结果	-	-	-	-
张明家	–		–		

检测方法与局限性

检测方法:

Sanger 测序

检测方法局限性:

适用于检测基因外显子及邻近±20bp 区域内的点突变及小的缺失插入突变。不适于检测基因调节区域和深度内含子区域的突变。不能检测染色体数目及结构异常、某些 DNA 大片段的缺失、DNA 大片段拷贝数变异以及动态突变、复杂重组等特殊类型突变。此外样本引物区域及测序引物区域发生突变、基因组多拷贝序列、复杂结构等因素均会影响检测的准确性。

注:

*本报告结果只对送检样品负责。本中心对以上检测结果保留最终解释权，如有疑义，请在收到结果后的7个工作日内与我们联系。以上结论为实验室检测数据，仅用于突变检测之目的，不代表最终诊断结果，仅供临床参考。由于疾病的复杂性和进展性以及每种检测技术方法的局限性，本次检测仅仅提供检测范围内的变异情况。委托方已充分了解该诊断研究的性质和风险，经本人及家属慎重考虑后同意接受实验分析，实验分析方已履行了告知义务。

文献

无

科睿迪医学

CRED DIAGNOSTICS

科睿迪医学

PRNP 基因检测

基本信息

**1.受检者**			
姓名：张胜方	性别：男	年龄：48	联系方式：-
**2.临床信息**			
诊断：-			
临床症状及家族史：-			
**3.样本及送检信息**			
样本编号：A00KRD034282	样本类型：☑EDTA 抗凝血	住院号/病历号：-	
送检医院：安徽中医药大学第一附属医院	科室：脑病科	床号：-	
采样日期：-	接收日期：2025-09-25	报告日期：2025-09-30	
**4.检测项目信息**			
检测项目：*PRNP* 基因测序			
检测方法：Sanger 测序			

检测结果

检测结论
在检测范围内，未检出致病或疑似致病变异<br>受检者检测到 129 位氨基酸多态性为 M/M 型，219 位氨基酸多态性为 E/E 型

检测结果统计

主要检测结果：与症状可能相关的变异

基因/转录本	基因亚区	突变信息	突变类型	致病性分类	遗传方式	疾病/表型	RS 号	文献
-	-	-	-	-	-	-	-	-

备注：
1.数据解读规则参考美国医学遗传学和基因组学学院（American College of Medical Genetics and Genomics，ACMG）相关指南。变异命名参照 HGVS 建议的规则给出（http://www.hgvs.org/mutnomen/）。
2.遗传方式：AD，常染色体显性遗传；AR，常染色体隐性遗传；XLD，X 染色体显性遗传；XLR，X 染色体隐性遗传；-，遗传方式未明。

本次检测，在受检者中未检出致病或疑似致病变异。

检验者：郭成忠　　报告者：吴引鸿

杭州科睿迪智检医学检验实验室 报告专用章 2025-9-30

检测基因列表

检测基因
PRNP

Sanger 测序结果

注：由于测序采用正向测序或反向测序，结果显示的碱基有可能为被检测碱基的反向互补序列，如：c.163G>A，峰图可显示为 G>A 或其反向互补序列 C>T。

分析样本	分析结果	-	-	-	-
张胜方	–		–		

检测方法与局限性

检测方法:
Sanger 测序

检测方法局限性:

适用于检测基因外显子及邻近±20bp 区域内的点突变及小的缺失插入突变。不适于检测基因调节区域和深度内含子区域的突变。不能检测染色体数目及结构异常、某些 DNA 大片段的缺失、DNA 大片段拷贝数变异以及动态突变、复杂重组等特殊类型突变。此外样本引物区域及测序引物区域发生突变、基因组多拷贝序列、复杂结构等因素均会影响检测的准确性。

注:

*本报告结果只对送检样品负责。本中心对以上检测结果保留最终解释权，如有疑义，请在收到结果后的7个工作日内与我们联系。以上结论为实验室检测数据，仅用于突变检测之目的，不代表最终诊断结果，仅供临床参考。由于疾病的复杂性和进展性以及每种检测技术方法的局限性，本次检测仅仅提供检测范围内的变异情况。委托方已充分了解该诊断研究的性质和风险，经本人及家属慎重考虑后同意接受实验分析，实验分析方已履行了告知义务。

文献

无

科睿迪医学
CRED DIAGNOSTICS
